# Comparative Study of Exchange Nailing and Augmentative Plating for Treating Aseptic Nonunion of Femoral Shafts Post Intramedullary Nailing: A Single-Blind, Multicentric Randomized Clinical Trial

**DOI:** 10.3390/jcm13226928

**Published:** 2024-11-18

**Authors:** Mehdi Motififard, Hamid Mousavi, Nasrollah Iranpanah, Hossein Akbari Aghdam, Mehdi Teimouri, Mohsen Aliakbari, Mohammad Parhamfar, Somaye Shirazi Nejad, Mahdi Shahsavan, Amin Daemi, Ashkan Salehi, Mohammad Shahsavan

**Affiliations:** 1 Department of Orthopedic Surgery, Isfahan University of Medical Sciences, Isfahan 81746-73461, Iranakbariaghdam@med.mui.ac.ir (H.A.A.);; 2 Department of Statistics, Faculty of Mathematics and Statistics, University of Isfahan, Isfahan 81746-73461, Iran; iranpanah@stat.ui.ac.ir; 3 Department of Radiology, Isfahan University of Medical Sciences, Isfahan 81746-73461, Iranmahdishah910@gmail.com (M.S.); 4 Department of Medical Biochemistry, Faculty of Medicine, Cukurova University, Adana 01330, Turkey; phd_bio@yahoo.com

**Keywords:** femoral fractures, fracture healing, intramedullary nailing, bone grafting, bone plates, treatment outcome

## Abstract

**Background**: Aseptic nonunion of femoral shafts after intramedullary nailing (IMN) can be a challenging condition that may lead to long-term disability and the need for multiple surgical procedures. This study compared the clinical and radiological outcomes between exchange nailing and augmentative plating with bone grafting. **Methods**: In this multicenter, prospective, single-blind, randomized controlled trial, patients with aseptic nonunion of the femoral shaft after IMN were randomly assigned to receive exchange nailing or augmentative plating. The primary outcomes measured were the time to bone union and union rate 12 months after revision surgery. The secondary outcomes included operative time, blood loss, hospitalization duration, pain level using the visual analog scale (VAS), knee range of motion (ROM), and complication rates. **Results:** The augmentative plating group had a significantly shorter mean time to union (5.39 ± 1.29 months) compared with the exchange nailing group (7.38 ± 1.97 months; *p* < 0.001). The union rates at 12 months were 100% in the augmentative plating group and 89.65% in the exchange nail group. Augmentative plating resulted in a shorter operation time (99.46 ± 11.08 min vs. 106.45 ± 12.22 min; *p* = 0.025) and reduced blood loss (514.79 ± 45.87 mL vs. 547.72 ± 54.35 mL; *p* = 0.016). Significant pain reduction was observed in the augmentative plating group, with preoperative VAS scores decreasing from 6.04 ± 2.28 to 2.64 ± 1.50, compared with a decrease from 5.66 ± 2.21 to 3.66 ± 2.19 in the exchange nailing group (*p* = 0.047). Knee ROM improved significantly in the augmentative plating group (*p* = 0.0176). The complication rate was lower in the augmentative plating group (3.57%) than in the exchange nail group (17.24%). **Conclusions**: Augmentative plating with autologous bone grafting is superior to exchange nailing for treating aseptic nonunion of femoral shafts. It is associated with faster healing, higher union rates, better clinical and functional outcomes, and fewer complications. We recommend this technique as the preferred treatment option for such complex cases.

## 1. Introduction

Femoral shaft fractures result from high-energy trauma, such as vehicular accidents or falls from a height [1]. Intramedullary nailing (IMN) is preferred in adult patients, in whom a metal rod is inserted into the femur to stabilize the bone, which offers several advantages, such as a high success rate, minimal invasiveness, and early weight-bearing support [2,3,4]. However, despite successful treatment, nonunion of the femoral shaft can occur after IMN [5]. This complication is characterized by inadequate bone healing and can significantly impede the recovery process [6]. 

The incidence of nonunion varies from 1% to 14%, depending on the type of fracture, surgical technique, and comorbidities such as diabetes and smoking [7,8,9,10,11]. Clinically, nonunion can be classified as septic or aseptic, and each type requires a different treatment approach [12,13]. 

Aseptic nonunion of the femoral shaft after IMN is particularly challenging, leading to prolonged disability and additional surgeries [5]. These nonunions significantly impair functional recovery and quality of life and impose economic burdens on healthcare systems [14]. Treating septic femoral shaft nonunion typically involves multiple surgeries, while aseptic femoral shaft nonunion can often be treated with a single surgical procedure [12,13]. 

Various treatment options exist for aseptic femoral shaft nonunion, including nail dynamization, exchange nailing, and plate augmentation [15]. Nail dynamization involves controlled movement at the fracture site by removing interlocking screws to promote healing [16,17,18]. However, its effectiveness is limited, particularly in comminuted fractures [16,19]. 

Nail exchange involves replacing an existing IMN with a larger diameter one to improve stability and promote healing by stimulating a biological response through reaming. This reaming process increases blood flow and activates cellular regeneration at the nonunion site [5,20]. In contrast, plate augmentation with autologous bone grafting provides mechanical stability and biological stimulation. The plate offers rigid fixation, minimizing micromovement at the fracture site, which is essential for successful bone healing. Additionally, the use of autologous bone graft supplies osteogenic cells and growth factors, creating an environment conducive to new bone formation and overall improved osteogenesis [21,22].

Several systematic reviews and meta-analyses, mainly based on retrospective cohort studies, have demonstrated that augmentative plating, compared to exchange nailing, is associated with increased bone union rates, shorter time to union, reduced intraoperative blood loss, and shorter operative durations in the treatment of aseptic femoral shaft nonunion following IMN [23,24]. While these findings suggest potential benefits with augmentative plating, the methodological limitations of the available evidence, including lack of randomization, retrospective designs, selection bias, and heterogeneity, preclude definitive conclusions on the optimal treatment approach [25,26,27]. 

To address this critical evidence gap, we conducted a multicenter, prospective, randomized controlled trial comparing augmentative plating with exchange nailing for aseptic femoral shaft nonunion following IMN. To our knowledge, this represents the first randomized comparative effectiveness trial evaluating radiological, clinical, functional, and safety outcomes between these two surgical techniques.

The primary outcome was time to radiographic union and postoperative union rate at 12 months. Secondary outcomes included perioperative factors, pain scores, range of motion, complications, and other clinically relevant endpoints. Based on the existing data, we hypothesized that augmentative plating would result in improved outcomes compared to exchange nailing. Findings from this robust randomized trial will provide definitive evidence to optimize clinical decision-making and improve outcomes for patients with aseptic femoral shaft nonunion. 

## 2. Materials and Methods

### 2.1. Study Design and Setting

This randomized controlled trial aimed to compare the clinical and radiological effectiveness of exchange nailing versus augmentative plating in treating aseptic femoral shaft nonunions following intramedullary nailing. The multicenter prospective study was conducted between November 2021 and March 2024 at the orthopedic departments of the Kashani Hospital and Al-Zahra Hospital, which serve as the major trauma referral centers in Isfahan, Iran. The single-blind design helped reduce potential researcher bias during outcome assessments. The Institutional Ethics Committee of the Isfahan University of Medical Sciences provided ethical approval for the study (approval code: IR.MUI.MED.REC.1400.624 on 8 November 2021), and it was registered with the Iranian Registry of Clinical Trials prior to patient recruitment (registration number: IRCT20230211057388N1).

### 2.2. Participants and Eligibility Criteria

This study involved individuals aged 18–65 diagnosed with aseptic nonunion of the femoral shaft following IMN. Nonunion was defined as the absence of bone healing at least six months after surgery, assessed through specific clinical and radiographic criteria [28,29]. Clinically, persistent pain was characterized by continuous discomfort at the fracture site during movement or weight-bearing activities, limiting mobility or range of motion [29,30]. Radiographic criteria for nonunion included the absence of bridging callus formation on at least three out of four cortices observed in anteroposterior and lateral radiographs, persistent fracture lines, increased bone sclerosis at the fracture edges, and no signs of progressive healing over time [8,31]. 

Fracture types were classified according to the AO/OTA classification system, encompassing types 32-A (simple fractures), 32-B (wedge fractures), and 32-C (complex fractures), focusing on the region between 5 cm below the lesser trochanter and 5 cm above the adductor tubercle [6,30]. Nonunion locations were further classified as non-isthmal (supra-isthmal or infra-isthmal) or isthmal based on their position relative to the femoral isthmus and distal femoral metaphyseal flare [30,32]. Nonunions were further classified radiographically as hypertrophic or atrophic. Hypertrophic nonunions exhibited excessive and disorganized callus formation due to inadequate mechanical stability, while atrophic nonunions showed minimal callus development resulting from insufficient vascular supply and limited biological activity [6,33].

We excluded patients with open fractures, pathological fractures, bilateral injuries, or severe comorbidities contraindicated for surgery. Additionally, individuals who could not adhere to scheduled follow-ups, presented with neurovascular dysfunction, or showed clinical signs of infection at the nonunion site were not eligible to participate. Nonunions were classified as aseptic or septic based on clinical and laboratory criteria. Aseptic nonunions were defined by the absence of clinical signs of infection, including local redness, swelling, purulent discharge, or sinus tract, normal white blood cell (WBC) counts, C-reactive protein (CRP) levels, and erythrocyte sedimentation rate (ESR). Additionally, there was no intraoperative evidence of purulence or positive cultures [5,28].

### 2.3. Randomization and Blinding

Patients were randomly assigned at a 1:1 ratio to either the exchange nailing or the augmentative plating group by an independent investigator using a computer-generated random block sequence. Each patient’s treatment assignment was placed securely in an opaque sealed envelope to ensure allocation concealment. After obtaining informed consent and completing the necessary admission procedures, each patient was formally enrolled and prepared for the surgery. The envelope was opened at this point to allow the surgical team to determine the treatment group assignment. This timing ensured that the team could perform the appropriate planning, equipment set-up, and patient positioning required for each procedure, as the two methods involved distinct materials and approaches. Patients remained unaware of their assigned treatment group throughout the study, minimizing bias in patient-reported outcomes such as pain levels and functional assessments. Outcome evaluations were conducted by independent assessors who were not involved in the surgeries or prior patient care. Although complete blinding of evaluators was not feasible due to the visible differences in interventions (e.g., the presence of a plate on radiographs), the assessors were not informed of the study’s hypotheses or aims, thus supporting objective and unbiased data collection. Informed consent was obtained from all participants prior to randomization, and they were fully informed about the surgical procedures, including their potential risks and benefits.

### 2.4. Interventions

Two experienced orthopedic surgeons (M.M. and H.M.) performed all interventions at each center using the same techniques under spinal or general anesthesia. The anesthesiologist determined the choice of anesthesia based on each patient’s clinical condition, medical history, individual preferences, and the anesthesiologist’s expertise. Prophylactic antibiotics were administered 30 min before the incision.

#### 2.4.1. Nail Exchange Technique

Exchange nailing was performed using a standardized surgical technique. The patient was placed in the supine position on a radiolucent table, and the affected limb was prepared and draped sterilely. The previous nail was extracted from the femoral canal under fluoroscopic guidance, using a nail extractor to minimize damage to the surrounding soft tissues and bone.

The canal was then prepared using progressively larger-diameter reamers to eliminate any remaining bone remnants and to encourage bleeding from the endosteal surface. Reaming was performed in 0.5 mm increments until the canal was reamed to 1–2 mm over the diameter of the new nail (Osveh Asia Medical Instrument Co., Mashhad, Iran). Attention should be paid to avoid excessive reaming, particularly in the proximal femur, to maintain stability and prevent iatrogenic fractures.

**Figure 1:**
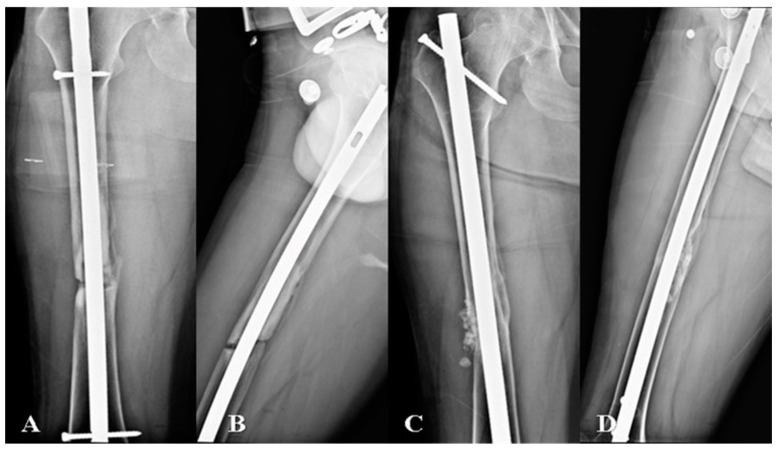
Radiological images showing a 34-year-old patient with atrophic nonunion of the femoral shaft who underwent exchange nailing. The images included preoperative radiographs: (**A**) anteroposterior radiograph and (**B**) lateral radiograph, as well as postoperative follow-up; (**C**) anteroposterior and (**D**) lateral radiographs.

The length and diameter of the new nail were determined based on the patient’s femoral anatomy and extent of reaming. A new IMN, 1–2 mm larger than the previous nail, was selected and assembled using the appropriate locking screws. The nail was inserted into the femoral canal in an antegrade manner through the pyriformis fossa or greater trochanter, depending on the initial surgery. The nail was then locked proximally and distally, using screws to provide rotational and axial stability [13,34]. The final fluoroscopic images were obtained to confirm nail position, fracture reduction, and locking screw placement (Figure 1).

#### 2.4.2. Plate Augmentation with Autologous Bone Grafting Technique

Surgery was performed with the patient in a supine position on a radiolucent table. The affected limb and ipsilateral hip were prepared and draped sterilely, with a sandbag placed under the affected limb. A direct lateral approach to the femur was used, centering the incision over the nonunion site. The tensor fascia lata and vastus lateralis muscles were split to expose the lateral aspect of the femur. Simultaneously, another team made a separate incision over the ipsilateral iliac crest to harvest the corticocancellous autogenous bone graft.

The nonunion site was identified, and the surrounding scar and fibrous tissues were carefully excised to expose the bone ends. The sclerotic bone at the fracture site was decorticated using a combination of osteotomes and curettes to create a bleeding bone surface and to promote healing. Care was taken to preserve as much viable bone as possible, while ensuring adequate decortication.

All patients underwent autogenous bone grafting. The graft was packed into the nonunion gap to promote bone formation and fixed with a plate and screws. A 4.5 mm AO dynamic compression plate (DCP) of appropriate length was selected based on the fracture location and anatomy (Osveh Asia Medical Instrument Co., Mashhad, Iran). The plate was contoured to match the lateral aspect of the femur and positioned over the nonunion site. At least three screws were placed on each side of the nonunion to achieve stable fixation. A combination of unicortical and bicortical screws was used, with unicortical screws preferred in areas where the IMN was present to prevent damage. No screws were placed directly into the nonunion site to prevent the disruption of the bone graft [22,33,35,36].

**Figure 2:**
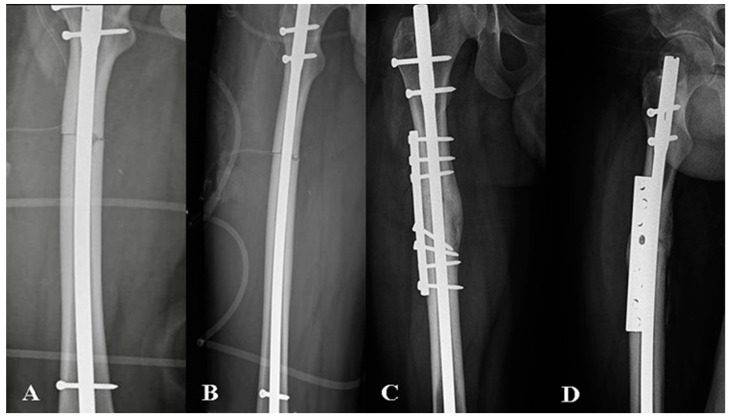
Radiological images depicting a 21-year-old patient with atrophic nonunion of the femoral shaft who underwent plate augmentation with an autologous bone graft. The images included preoperative radiographs: (**A**) anteroposterior radiograph and (**B**) lateral radiograph, as well as postoperative follow-up; (**C**) anteroposterior and (**D**) lateral radiographs.

The final fluoroscopic images were obtained to confirm the plate position, screw placement, and fracture alignment (Figure 2). The wound was thoroughly irrigated, and the soft tissues were closed in layers over the surgical drain to prevent hematoma formation. 

### 2.5. Data Collection and Outcome Measures

Demographic and clinical data were collected prospectively at baseline from all participants, encompassing factors such as age, sex, body mass index (BMI), smoking status, and the mechanism of injury, categorized as high-energy or low-energy trauma. The characteristics of nonunion, whether hypertrophic or atrophic, and its location (supra-isthmal, isthmal, or infra-isthmal) were also documented. Furthermore, fractures were classified according to the AO/OTA system, and the interval between the initial fracture fixation and the subsequent revision surgery was recorded. Comorbidities, including diabetes mellitus and hypertension, among others, were noted. BMI was calculated by dividing weight in kilograms by height in meters squared (kg/m^2^). Smoking status was assessed through structured interviews with participants, classifying them as current smokers or non-smokers based on their self-reported habits.

The primary outcomes were the time to bone healing and union rate within 12 months after revision surgery. Time to union was defined as the period required for bone healing, assessed by the presence of a bridging callus on three of the four cortices on anteroposterior and lateral radiographs. The union rate was calculated as the number of patients who achieved bone healing within 12 months divided by the total number of patients in each group.

The secondary outcomes included operative metrics, such as operative time, blood loss, and hospitalization duration. The operative time was measured from skin incision to closure. Blood loss was recorded as the total volume lost during surgery, and hospitalization duration was the length of stay post-surgery until discharge. Clinical and functional assessments included pain level, knee range of motion (ROM), and complications. Active knee flexion ROM was measured using a goniometer, while pain levels were assessed using the Visual Analog Scale (VAS) preoperatively and 12 months postoperatively. Documented complications included infections, malalignment, deep vein thrombosis, loose or broken internal fixation, and failure to achieve bone healing within 12 months [37]. Angular and rotational alignments were measured radiographically, with malalignment defined as a 5° angulation, 15° malrotation, and 2 cm length discrepancy [38]. 

### 2.6. Postoperative Care and Follow-Up

Postoperative care was tailored to each patient’s clinical and radiographic progress. All patients initially received standardized care, including thromboprophylaxis with low molecular weight heparin for four weeks and knee ROM exercises starting on the second postoperative day. Toe-touch weight-bearing with a walking device began on day three. Progression to partial and full weight-bearing was guided by clinical and radiologic assessments, including pain levels, functional capacity in physical therapy, and callus formation seen on anteroposterior and lateral radiographs at four to six weeks. Patients with delayed healing or persistent pain maintained protected weight-bearing and modified rehabilitation. Follow-up evaluations, including clinical and radiographic assessments, were conducted every six weeks to monitor healing and adjust care as needed. 

### 2.7. Statistical Analysis

The data were analyzed using SPSS software version 26 (SPSS Inc., Chicago, IL, USA). Descriptive statistics were used to summarize the demographic characteristics. The mean ± standard deviation (SD) was used for continuous variables, whereas frequencies and percentages were used for categorical variables. The normality of the data distribution was assessed using the Shapiro-Wilk test. An independent *t*-test was used to compare the means between groups for normally distributed variables, and the Mann–Whitney U test was used for non-normally distributed variables. Categorical variables were analyzed using the standard chi-squared test. The Wilcoxon signed-rank test was used to compare the VAS scores and knee ROM changes within each group before and after surgery. A significance level of *p* < 0.05 was considered statistically significant for all analyses. Missing data were not imputed, and a complete case analysis was performed. 

### 2.8. Sample Size Calculation

The sample size was calculated using the following formula to compare the two proportions:

n=(Z1−α2+Z1−β)2×P11−P1+P21−P2P1−P22

where:

Z1−α2=1.96 (Z−score for a 95% confidence level)

$$Z_{1-\beta }=1.28(Z-score\;for\;90\%\;power)$$

α=0.05 (significant level)

β=0.1 (type II error probability)

$$P_{1}=0.2\;(proportion\;in\;the\;nail\;exchange\;group)$$

$$P_{2}=0.6\;(proportion\;in\;the\;plate\;augmentative\;group)$$

The assumed proportions, $P_{1}$ and $P_{2}$, were determined based on previous studies and expert input in mathematics and statistics (N.I.) [15,36,37,39]. Using these values, the formula indicates that a sample size of at least 26 participants per study group is required. This sample size ensures that the study will have 90% power to detect a statistically significant difference between the two proportions at a 5% significance level, assuming the accurate proportions match the assumed values. This calculation ensures adequate power to test the hypothesis effectively.

## 3. Results

### 3.1. Participant Flow

**Figure 3:**
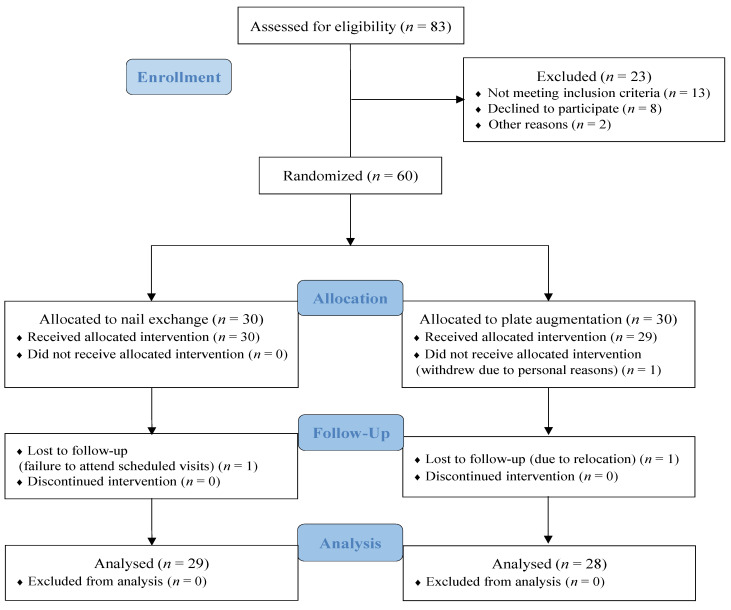
CONSORT flow diagram of the study participants.

A total of 83 patients with aseptic nonunion of the femoral shaft were initially screened for eligibility. Of these, 13 patients did not meet the inclusion criteria, eight declined to participate, and two were excluded for other reasons. Sixty eligible patients were randomly assigned to the following treatment groups: nail exchange (*n* = 30) and plate augmentation with bone grafting (*n* = 30). During the follow-up period, two patients were lost to follow-up in the plate augmentation group (*n* = 28) and one patient was lost to follow-up in the nail exchange group (*n* = 29). The CONSORT diagram visually represents the participant flow throughout the study (Figure 3).

### 3.2. Demographic Characteristics

The participants’ demographics were well-matched between the two groups. The nail exchange group had an average age of 38.07 ± 11.49 years, while the plate augmentation group averaged 36.32 ± 10.42 years (*p* = 0.550). The mean BMI was similar in both groups, with 28.30 ± 1.91 kg/m^2^ in the nail exchange group and 28.29 ± 1.23 kg/m^2^ in the plate augmentation group (*p* = 0.982). Sex distribution showed that males predominated in both groups, with 79.31% and 85.71% in the nail exchange and plate augmentation groups, respectively (*p* = 0.544) (Table 1).

### 3.3. Descriptive Characteristics 

In the nonunion locations, isthmal nonunions were most common in the nail exchange group (68.97%), whereas supra-isthmal nonunion was more common in the plate augmentation group (35.71%). Infra-isthmal nonunion appeared least frequently in both groups (17.24% and 25.00%, respectively; *p* = 0.064). Regarding fracture characteristics, fractures were classified according to the AO/OTA system. In the nail exchange group, 44.83% were type 32-A (simple fractures), 31.03% were type 32-B (wedge fractures), and 24.14% were type 32-C (complex fractures). In the plate augmentation group, 25.00% were type 32-A, 28.57% were type 32-B, and 46.43% were type 32-C. However, this difference was not statistically significant (*p* = 0.162).

The primary cause of injury in both groups was high-energy trauma, primarily vehicular accidents, accounting for 82.76% of cases in the nail exchange group and 67.86% in the plate augmentation group (*p* = 0.318). Smoking status was recorded, with 27.59% of the nail exchange group and 17.86% of the plate augmentation group identified as current smokers, although this difference was not statistically significant (*p* = 0.576). Additionally, comorbidities such as diabetes mellitus and hypertension were more prevalent in the nail exchange group (41.38%) compared to the plate augmentation group (28.57%; *p* = 0.462). 

Both groups had a higher proportion of atrophic nonunions, with a slightly greater prevalence in the plate augmentation group (64.29% vs. 51.72%; *p* = 0.489) (Table 2). 

### 3.4. Primary Outcomes 

#### 3.4.1. Time to Union

The plate augmentation group showed a notably shorter mean time to union of 5.39 ± 1.29 months, while the exchange nailing group exhibited a significantly longer mean time to union of 7.38 ± 1.97 months (95% CI: 1.07, 2.92; *p* < 0.001).

#### 3.4.2. Union Rate

The plate augmentation group demonstrated a union rate of 100% (28/28) at the 12-month post-revision surgery mark. In comparison, the exchange nailing group had a union rate of 89.65% (26/29).

### 3.5. Secondary Outcomes

#### 3.5.1. Perioperative Outcomes

Patients undergoing exchange nailing experienced a significantly longer mean operative time of 106.45 ± 12.22 min compared to 99.46 ± 11.08 min for those receiving augmentative plating (*p* = 0.025). Intraoperative blood loss was significantly higher in the exchange nailing group at a mean of 547.72 ± 54.35 mL versus 514.79 ± 45.87 mL in the plate augmentation cohort (*p* = 0.016). The mean postoperative length of hospital stay did not differ significantly between the two groups (*p* = 0.580). Furthermore, the interval between the initial femoral shaft fracture fixation and the subsequent revision surgery for nonunion was similar in both groups (*p* = 0.836) (Table 3).

#### 3.5.2. Comparative Analysis of Pain Levels 

**Figure 4:**
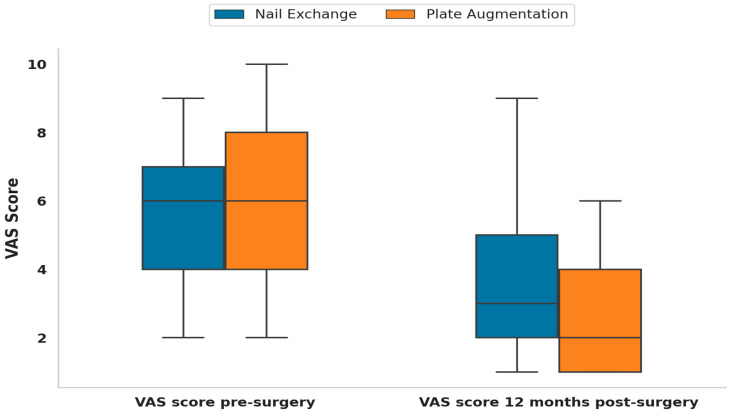
The plate augmentation group showed a significant reduction in pain level at the 12-month follow-up, demonstrating superior functional recovery.

The analysis involved comparing VAS scores before surgery and 12 months after surgery, and the changes in VAS scores between the nail exchange and plate augmentation groups. Before surgery, the mean VAS score for the nail exchange group was 5.66 ± 2.21; for the plate augmentation group, it was 6.04 ± 2.28. A Wilcoxon signed-rank test revealed a statistically significant reduction in pain levels within each group (*p* < 0.0001). Twelve months post-surgery, the mean VAS score for the nail exchange group was 3.66 ± 2.19, while for the plate augmentation group, it was 2.64 ± 1.50. An independent *t*-test demonstrated a statistically significant difference between the two groups at this time point (*p* = 0.047). However, the mean change in VAS score was −2.00 ± 1.53 for the nail exchange group and −3.40 ± 1.83 for the plate augmentation group, with the independent *t*-test revealing a statistically significant difference in the changes between the groups (*p* < 0.001) (Table 4). These results suggest a significant improvement in pain levels within each group over time, with a more significant reduction in pain observed in the plate augmentation group than in the nail exchange group (Figure 4).

#### 3.5.3. Knee Function Assessment

**Figure 5:**
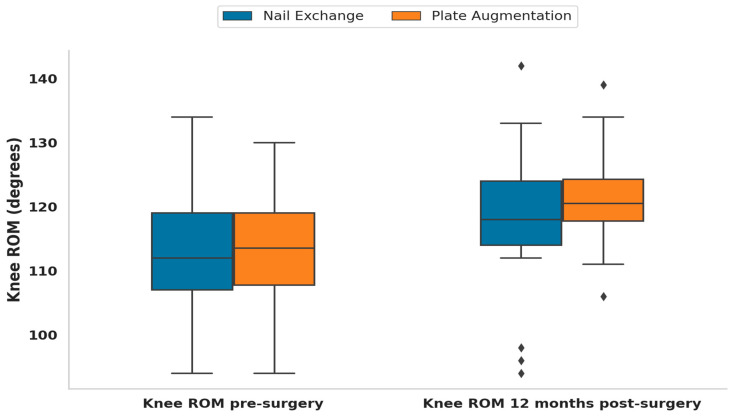
The plate augmentation group exhibited slightly better improvement in knee flexion at the 12-month follow-up, demonstrating its effectiveness in restoring joint function and mobility.

In this study, knee ROM was compared before and 12 months after surgery, as were the changes in knee ROM between the nail exchange and plate augmentation groups. Before surgery, the mean knee ROM was 111.69° ± 10.28° in the nail exchange group and 113.11° ± 8.3° in the plate augmentation group. The Wilcoxon signed-rank test revealed a statistically significant difference between the groups (*p* < 0.0001). Twelve months after surgery, the mean knee ROM in the nail exchange group was 118.14° ± 10.37°, whereas that in the plate augmentation group was 121.36° ± 7.03°. The Mann–Whitney U test showed no statistically significant difference between the two groups at this time point (*p* = 0.1285). However, the mean change in knee ROM was 6.45° ± 3.63° for the nail exchange group and 8.25° ± 3.92° for the plate augmentation group, with a statistically significant difference between the groups (*p* = 0.0176) according to the Mann Whitney U test (Table 5). These results suggest notable improvements within each group over time, with more significant improvements observed in the plate augmentation group than in the nail exchange group (Figure 5).

#### 3.5.4. Complications

During the 12-month follow-up, 96.43% of the plate augmentation group encountered no complications, with 3.57% reporting superficial infections. In the exchange nailing group, 82.76% experienced no complications, 10.34% reported persistent nonunion, and 6.90% reported superficial infections. Notably, the association between the treatment type and complication rate was not statistically significant (*p* = 0.174). No malalignment, deep vein embolism, loose internal fixation, or breakage was reported postoperatively.

## 4. Discussion

This randomized controlled trial provides valuable evidence that both exchange nailing and augmentative plating effectively treat aseptic femoral shaft nonunions following IMN. However, the results indicate that plate augmentation achieves superior clinical and radiological outcomes compared with nail exchange. Specifically, the plate augmentation cohort demonstrated a significantly shorter mean time to union of 5.39 months versus 7.38 months with nail exchange (*p* < 0.001). Additionally, plate augmentation achieved a 100% union rate at 12 months, whereas the union rate was 89.65% with nail exchange. 

The significantly shorter time to union and higher union rate observed in the augmentative plating group provide compelling evidence that this surgical technique is more effective in stimulating bone healing in patients with aseptic femoral shaft nonunion after IMN. These findings support the initial hypothesis that augmentative plating with autologous bone grafting would yield superior outcomes compared to exchange nailing in this clinical context. The success of augmentative plating can be attributed to the synergistic effect of the biological stimulus and the mechanical stability of the autologous bone graft [22,40].

Autologous bone grafts are the gold standard for bone regeneration, providing essential osteogenic cells, osteoconductive scaffolds, and osteoinductive growth factors [41,42]. The graft stimulates new bone formation at the nonunion site by promoting osteogenesis and providing a conducive environment for osteoblast differentiation and activity, thereby enhancing the biological circumstances at the nonunion site and facilitating faster bone consolidation [6,35,43]. The mechanical stability of the plate further enhances this biological stimulus, ensuring that the graft remains in place and is subjected to appropriate mechanical forces to facilitate bone healing [10,13]. Dynamic compression plating (DCP), leaving the nail in situ, provides consistent and stable compression, which is more effective in maintaining alignment and promoting union [36,40,44,45]. In our research, we utilized a DCP for plate augmentation in treating aseptic femoral shaft nonunion, achieving a 100% union rate within an average duration of 5.39 ± 1.29 months. Perisano et al. [6] examined the literature on aseptic femoral shaft nonunion following IMN, encompassing 24 studies with 502 patients. The analysis revealed that DCP was the most commonly employed plate for plate augmentation, with 87.1% of the cases also incorporating a bone graft. Complete bone healing was attained in 98.0% of the patients, with an average duration of 5.8 ± 2.12 months.

The unique combination of mechanical stability, achieved by combining the rigid fixation of a plate with the existing IMN, and biological factors creates a more stable construct that supports bone healing more effectively than a larger-diameter nail alone, as demonstrated by the superior results achieved with augmentative plating in this study. Numerous studies have highlighted the importance of integrating bone grafts in nonunion treatment, as it significantly improves healing outcomes, underscoring the importance of biological augmentation in conjunction with mechanical stabilization [42,46,47].

The findings of this study align with the existing literature, particularly the recent study in 2023 by Walcher et al. [48], which emphasized the effectiveness of plate augmentation in creating a more cohesive approach to fracture stabilization. This technique improves the stability of the fracture site, enhances the overall construct stability, and promotes bone healing. Additionally, Lai et al. [47] noted that augmentative plating effectively addresses rotational instability compared to exchange nailing, resulting in reduced mechanical failure and an increased likelihood of bone healing.

In a biomechanical study conducted by Ma et al. [49], plate augmentation was used in the treatment of femoral shaft nonunion following IMN. The study concluded that using three screws on each side, rather than two, resulted in improved rotational stability. Additionally, the study found no significant difference in stability between single cortical screw fixation and bicortical screw fixation when the number of screws was consistent. The study recommended the use of at least three single cortical locking or bicortical screws on each side. Our study aligned with these recommendations, incorporating a minimum of three cortical screws on each side of the plate whenever possible. Bicortical screws were used when feasible, with unicortical screws being preferred in areas where the IMN was present to prevent damage to the nail.

In contrast, the exchange nailing group showed a longer time to union and lower union rate, which may be due to the limited biological stimuli provided by this technique. Exchange nailing mainly relies on the reaming process to stimulate bone growth, which may not always be effective in cases of nonunion. This deficiency in biological stimuli could explain the suboptimal outcomes observed in the exchange nail group [10]. In addition, Uliana et al. [21] proposed that reaming the IM canal to accommodate a larger nail may damage the endosteal blood vessels, consequently affecting the biological healing response.

During this study, it was observed that patients who underwent exchange nailing experienced longer operative durations and increased blood loss. These findings could be attributed to the technical complexities involved in removing the existing nail, inserting a new nail, and the extensive reaming process [11]. Additionally, exchange nailing requires more frequent radiography than plate augmentation, contributing to longer operative times. Prior research has shown that plate augmentation results in lower intraoperative blood loss than exchange nailing [11,47,50]. Minimizing blood loss is crucial, as it reduces the risk of postoperative complications, such as anemia and hemorrhagic shock, which can negatively impact bone healing and overall functional recovery [47].

The analysis of VAS scores provides valuable insights into the efficacy of pain management in treating femoral shaft nonunion with nail exchange and plate augmentation. Both groups initially reported similar levels of pain; however, the post-surgery outcomes revealed a more significant reduction in pain in the plate augmentation group. These findings suggested that plate augmentation facilitates bone healing and provides superior pain relief. The statistically significant improvement in pain reduction in the plate augmentation group supports the argument that this method is more effective in managing postoperative pain, which is further supported by studies that emphasize the benefits of this technique in reducing long-term discomfort [51,52,53]. The improved pain relief observed with plate augmentation may be attributed to the more robust mechanical stability provided by the plates, which minimizes micromovements at the fracture site and consequently reduces pain stimuli [52,54,55].

Postoperative knee ROM significantly improved in both groups (*p* < 0.001). However, the plate augmentation group exhibited slightly better knee flexion at the 12-month follow-up. This suggests that both techniques restore joint function, likely because of pain resolution and enhanced bone stability post-intervention. These findings are consistent with those of previous studies indicating that patients who undergo augmentative plating experience significant improvements in joint function and patient recovery [13,40,44,46,56,57,58]. Flowers et al. [59] noted that patients treated with augmentation plating typically allowed partial or full weight-bearing sooner than those treated with exchange nailing. Early weight-bearing has been demonstrated to enhance functional outcomes and accelerate the healing process [60]. 

These findings underscore the importance of clinicians carefully considering the benefits of pain reduction and functional recovery when choosing between nail exchange and plate augmentation for treating nonunion femoral shafts.

Our study revealed that the nail exchange group had a higher complication rate (17.24%) than the plate augmentation group (3.57%). In the nail exchange group, three patients did not experience bone healing (re-nonunion) during the 12-month follow-up, requiring further surgical intervention. In contrast, all the patients who underwent plate augmentation achieved a 100% union rate. Additionally, although two patients in the nail exchange group and one in the plate augmentation group experienced superficial infections, they were effectively managed with oral antibiotics and did not require additional surgical procedures. These findings highlight the superior safety profile of plate augmentation. The lower complication rates associated with augmentative plating demonstrate its potential to improve patient care and overall quality of life [42]. Consistent with our results, a meta-analysis demonstrated that plate augmentation resulted in a lower complication rate during the postoperative period and highlighted the negative impact of complications, including superficial infection and re-nonunion, on fracture healing and increased hospital costs [11].

Our study found no significant differences in factors such as smoking, comorbidities, and nonunion characteristics between the two groups. However, previous research has suggested the potential benefits of augmentative plating for nonunion characteristics [21,39], and we believe that this approach may be suitable across a range of patient-related factors and different types of nonunion. According to Ru et al. [50], plate compression augmentation demonstrates broader indications and a heightened union rate compared to the prevalent exchange nailing treatment, particularly for non-isthmal femoral shaft nonunions or isthmal cases [61]. Nevertheless, further investigations are warranted to establish the correlations between these variables and the outcomes of each surgical procedure.

The implications of this study suggest that augmentative plating should be considered the primary treatment for patients with aseptic femoral shaft nonunions rather than nail exchange due to its significant clinical benefits. This method optimizes functional recovery and improves patient outcomes by providing superior mechanical stability and biological stimulation through autologous bone grafting. Consequently, patients can resume weight-bearing activities and daily routines sooner, reducing the disability associated with prolonged nonunion. Lower complication rates, such as persistent nonunion and infections, underscore the clinical efficacy and reliability of augmentative plating over the nail exchange approach. 

However, the study has several limitations. The 12-month follow-up period restricts the evaluation of long-term outcomes. Extended follow-up is essential to assess the durability of the union, potential implant-related issues, and sustained functional improvements. Future research should investigate whether these short-term benefits translate into long-term recovery, return to work, and quality of life improvements. The study’s generalizability is constrained, as it was conducted in two trauma centers within one country, which may affect the applicability of the results to other settings with different demographics and healthcare systems. Furthermore, the lack of validated functional outcome measures, such as the Lower Extremity Functional Scale (LEFS) or Short Form-36 (SF-36), impacts the comprehensive evaluation of patient function and quality of life. The involvement of different surgical teams could have caused potential biases despite standardized protocols.

Future studies should address these limitations by incorporating longer follow-up periods, larger and more diverse populations, validated functional outcome measures, and cost-effectiveness analyses to evaluate the economic implications of augmentative plating compared to exchange nailing.

## 5. Conclusions

This randomized controlled trial provides compelling evidence that augmentative plating with autologous bone grafting is superior to exchange nailing for treating aseptic nonunion of femoral shafts following intramedullary nailing. The augmentative plating technique demonstrated significant advantages in terms of faster time to union, higher union rates, reduced operative times, decreased blood loss, enhanced pain relief, and improved post-surgical knee movement. Moreover, this method exhibited fewer complications, including re-nonunion and infections, than exchange nailing. These findings support augmentative plating as the preferred approach for managing this challenging condition, potentially leading to improved patient outcomes and reduced disability.

## Figures and Tables

**Table 1:** Demographic characteristics of the study participants.

Variables	Nail Exchange (N = 29) (Mean ± SD/%)	Plate Augmentation (N = 28) (Mean ± SD/%)	95% CI (Difference)	*p*-Value
Age (year)	38.07 ± 11.49	36.32 ± 10.42	(−4.08, 7.58)	0.550 ^a^
BMI (kg/m^2^)	28.30 ± 1.91	28.29 ± 1.23	(−0.85, 0.87)	0.982 ^a^
Gender (F/M)	20.69/79.31	14.29/85.71	(−0.10, 0.30)/(−0.30, 0.10) ^c^	0.544 ^b^

^a^ Independent *t*-test; ^b^ Chi-square; ^c^ 95% Confidence intervals for differences in proportions.

**Table 2:** Descriptive characteristics of the study participants.

Variables	Sub-Categories	Nail Exchange (%)	Plate Augmentation (%)	*p*-Value ^a^
Nonunion location	Supra-isthmal	13.79	35.71	0.064
	Isthmal	68.97	39.29	
	Infra-isthmal	17.24	25.00	
Fracture classification	32-A	44.83	25.00	0.162
	32-B	31.03	28.57	
	32-C	24.14	46.43	
Comorbidities	Yes	41.38	28.57	0.462
	No	58.62	71.43	
Mechanism of injury	High-energy	82.76	67.86	0.318
	Low-energy	17.24	32.14	
Smoking status	Yes	27.59	17.86	0.576
	No	72.41	82.14	
Type of nonunion	Hypertrophic	48.28	35.71	0.489
	Atrophic	51.72	64.29	

^a^ Chi-square test.

**Table 3:** Surgical outcomes and metrics.

Variables	Nail Exchange (N = 29) (Mean ± SD)	Plate Augmentation (N = 28) (Mean ± SD)	95% CI (Differences)	*p*-Value
Operation time (min)	106.45 ± 12.22	99.46 ± 11.08	(0.80, 13.17)	0.025 ^a,b^
Hospitalization (day)	4.83 ± 0.66	4.75 ± 0.80	(−0.31, 0.47)	0.580
Time to revision surgery (month)	12.24 ± 1.55	12.14 ± 1.24	(−0.65, 0.85)	0.836
Blood loss (ml)	547.72 ± 54.35	514.79 ± 45.87	(6.20, 59.68)	0.016 ^b^

^a^ Mann–Whitney U test; ^b^ Significant variables (*p* < 0.05).

**Table 4:** Comparative analysis of VAS scores before and 12 months after the surgical procedures.

Metrics	Nail Exchange (Mean ± SD)	Plate Augmentation (Mean ± SD)	*p*-Value (Within Group)	*p*-Value (Between Groups)	*p*-Value (At 12 Months)
VAS scores pre-surgery	5.66 ± 2.21	6.04 ± 2.28	<0.0001 ^a,c^		
VAS scores post-surgery	3.66 ± 2.19	2.64 ± 1.50	<0.0001 ^a,c^		0.047 ^b,c^
Change in VAS score	−2.00 ± 1.53	−3.40 ± 1.83		<0.001 ^b,c^	

^a^ Wilcoxon signed-rank test; ^b^ Independent *t*-test; **^c^** Significant variables (*p* < 0.05).

**Table 5:** Comparative analysis of the surgical techniques affecting knee function.

Metrics	Nail Exchange Mean ± SD	Plate Augmentation Mean ± SD	*p*-Value (Within Group)	*p*-Value (Between Groups)	*p*-Value (At 12 Months)
Knee ROM pre-surgery	111.69 ± 10.28	113.11 ± 8.31	<0.0001 ^a,c^		
Knee ROM post-surgery	118.14 ± 10.37	121.36 ± 7.03	<0.0001 ^a,c^		0.128 ^b^
Change in knee ROM	6.45 ± 3.63	8.25 ± 3.92		<0.017 ^b,c^	

^a^ Wilcoxon signed-rank test; ^b^ Mann–Whitney U test; ^c^ Significant variables (*p* < 0.05).

## Data Availability

The raw data supporting the conclusions of this article will be made available by the authors on request.
